# Virtual reality therapy targeting ideas of reference in patients with psychosis: a single-blind parallel-group randomized controlled trial

**DOI:** 10.1017/S0033291725000959

**Published:** 2025-04-28

**Authors:** EunJin Jeon, Ling Li, Thi-Hung Le, Woo-Sung Kim, Soyolsaikhan Odkhuu, Chae Yeong Kang, Ariana Setiani, Fatima Zahra Rami, Young-Chul Chung

**Affiliations:** 1 Department of Psychiatry, Jeonbuk National University Medical School, Jeonju, Republic of Korea; 2 Research Institute of Clinical Medicine, Jeonbuk National University-Biomedical Research Institute of Jeonbuk National University Hospital, Jeonju, Republic of Korea

**Keywords:** ideas of reference, path analysis, psychosis, virtual reality therapy

## Abstract

**Background:**

No studies have investigated the effects of virtual reality (VR) on the persecutory idea of reference (IOR) or delusions of reference (DOR) in patients with psychosis. This study examined the efficacy and safety of VR therapy in stable outpatients with psychosis and explored relationships between primary outcomes and psychological factors using path analysis.

**Methods:**

Seventy-eight patients were randomly assigned to either the VR-treatment (VR-T) or VR-control (VR-C) group. The VR-T group viewed three 360° 3D videos or four animated videos; the VR-C group viewed the same seven videos with muted voices or 11 360° 3D videos of natural scenes. Pre- and post-assessments were performed using the Psychotic Symptom Rating Scale-Delusions (PSYRATS-D) and Revised Green et al. Paranoid Thought Scale (R-GPTS) as a primary outcome measure. Several self-rating scales measuring schema, depression, brooding, negative evaluation, attribution bias, and self-esteem were administered. Safety was assessed after sessions 1 and 10, and path models were constructed.

**Results:**

Between-group analysis showed a significant improvement in PSYRATS-D scores in the VR-T group compared with the VR-C group. Regarding self-rating scales, the between-group analysis revealed a significant group × time interaction only for the Social and Occupational Functioning Assessment Scale (SOFAS) score. The frequency of VR sickness was high, but its severity was mild. Fear of Negative Evaluation Scale and Beck Depression Inventory scores were found to have mediating roles.

**Conclusions:**

VR therapy effectively reduced delusions in young, stable psychosis patients with mild and tolerable side effects. Future studies should develop diverse VR content for older populations.

## Introduction

Ideas of reference (IOR) involve self-attributions about events in one’s surroundings, particularly in social contexts, where unimportant stimuli are interpreted as being directed at oneself (Colori, 2017). IOR, among the most common symptoms of psychotic disorders (World Health Organization, 1973), also are observed in various other conditions, including mood and personality disorders (Colori, 2017; Meyer & Lenzenweger, 2009). They often emerge during the prodromal period of psychosis (Bendala-Rodríguez, Senín-Calderón, Peluso-Crespi, & Rodríguez-Testal, 2019) and appear in the residual phase of schizophrenia spectrum disorders (SSDs) (Schennach et al., 2015). Many stable outpatients with psychosis report ongoing IOR of a persecutory nature and mild persecutory delusions. Psychological factors or states linked to the development of IOR and persecutory delusions include attributional bias (Kaney & Bentall, 1989; Kinderman & Bentall, 1996), depression (Rodríguez-Testal, Bendala-Rodríguez, Perona-Garcelán, & Senín-Calderón, 2019), low self-esteem (Cicero & Kerns, 2011), negative beliefs about the self and others (Freeman et al., 2002; Lyon, Kaney, & Bentall, 1994), rumination (Wong et al., 2021), and social-evaluative concerns (Freeman et al., 2005). A comprehensive understanding of how these factors interact with each other using path analysis might help in targeting core symptoms in the treatment of patients with IOR.

Virtual reality (VR) has been identified as a potentially revolutionary tool for treating mental disorders. VR can be used to trigger cognitive, emotional, physiological, and behavioral responses to simulated real-life situations (Martens et al., 2019) while offering consistent, controlled, graded, repetitive, and systematic therapeutic training (Freeman et al., 2017). In the field of psychosis, the safety and acceptability of VR were initially tested (Fornells-Ambrojo et al., 2008; Valmaggia et al., 2007), followed by evaluations of its effects on cognition and symptoms (Fajnerová et al., 2014; Ruse et al., 2014; Veling et al., 2016). The application of VR has gradually expanded to cognitive remediation, social skills training, vocational training, and the treatment of delusions and auditory hallucinations (Rus-Calafell et al., 2018). Seven studies have used VR therapy to manage persecutory delusions, paranoia, and agoraphobic avoidance (Dellazizzo, Potvin, Phraxayavong, & Dumais, 2021; Freeman et al., 2016, 2022; Gega et al., 2013; Geraets et al., 2020; Moritz et al., 2014; Pot-Kolder et al., 2018). However, only four of those studies were randomized controlled trials (RCT) involving multiple sessions. Among these, two measured IOR by using the Green et al. Paranoid Thought Scale (GPTS) (Pot-Kolder et al., 2018) or Revised Green et al. Paranoid Thought Scale (R-GPTS) (Freeman et al., 2022). The former observed significant improvement of IOR with 16 sessions but the latter no improvement with 6 sessions. It is of note that the baseline score of the GPTS in the former was modestly high and the score of the R-GPTS in the latter was low and similar to ours. In Dellazizzo et al. study (2021), the target subjects were patients with treatment-resistant schizophrenia and 9 sessions were provided. In Geraets et al. study (Geraets et al., 2020), although patients were recruited with the inclusion criteria of GPTS >40, the outcome measure was an experience sampling method, and 16 sessions were performed. All three non-RCT employed one session. Although Gega et al. study (Gega et al., 2013) used GPTS, they reported only the total score and the number of participants was 6. Several VR studies conducted in Asian countries have mainly focused on social skills and vocational training (Chan, Ngai, Leung, & Wong, 2010; Ku et al., 2007; Park et al., 2011; Tsang & Man, 2013); only one investigated VR-based mindfulness training (Lee et al., 2023). Based on the review of relevant literature, we hypothesized that VR therapy with longer sessions would lead to a reduction in IOR or delusion of reference (DOR) in stable outpatients with psychosis and its therapeutic effects could be mediated by the influence on the psychological factors mentioned above.

The present study aimed to evaluate the efficacy and safety of VR treatment (VR-T) for persecutory IOR and DOR in stable outpatients with psychosis. The primary outcome measures were Psychotic Symptom Rating Scale-Delusions (PSYRATS-D) and Revised Green et al. Paranoid Thought Scale (R-GPTS). In addition, path analysis was conducted to examine direct, indirect, and mediation effects related to the primary outcome measures and pre-defined psychological factors (attributional bias, depression, low self-esteem, negative beliefs, rumination, and social-evaluative concerns).

## Methods

### Participants

Patients were recruited from six psychiatric outpatient clinics – Jeonbuk National University Hospital, Kyungpook National University Hospital, Chonnam National University Hospital, Catholic University of Korea Seoul St. Mary’s Hospital, Yeungnam University Medical Center, and Inje University Haeundae Paik Hospital – between August 2022 and May 2024. The inclusion criteria were as follows: diagnosis of an SSD (schizophrenia, schizoaffective disorder, or schizophreniform disorder) or another psychotic disorder (other specified schizophrenia spectrum and other psychotic disorders, and unspecified schizophrenia spectrum and other psychotic disorders), age 15–59 years, and stable outpatient status with no medication changes in the preceding 2 months. Diagnoses were based on the criteria of the Diagnostic and Statistical Manual of Mental Disorders, Fifth Edition (American Psychiatric Association & Association, 2013). The exclusion criteria were as follows: intelligence quotient ≤70; history of head trauma; acute, unstable, or severe medical/neurological condition; and pregnancy or lactation. Experienced psychiatrists from each institute evaluated the final diagnosis and invited patients to participate in the study. Initially, 90 individuals who met the criteria were randomly approached, and 12 declined to participate. The remaining 78 patients were randomly assigned to the VR-T or VR-control (VR-C) groups using a validated online randomizer (https://www.randomizer.org/). The authors affirm that all procedures in this study adhere to the ethical standards of the relevant national and institutional review boards and the 1975 Declaration of Helsinki (as amended in 2008). All procedures involving human participants were approved by the Ethics Committee of Jeonbuk National University Hospital (approval number: CUH 2022-04-018). The trial was registered with the Clinical Research Information Service (KCT0007710).

### Measures

Sociodemographic data (sex, age, age of onset, and education), clinical information (duration of illness and diagnosis), and medication data were collected by research nurses via chart review and psychiatrists reevaluated. The total daily dose of antipsychotics at baseline was converted to chlorpromazine equivalents based on the defined daily dose (Leucht, Samara, Heres, & Davis, 2016). Psychopathology and functioning were assessed using the Positive and Negative Syndrome Scale (PANSS) (Kay, Fiszbein, & Opler, 1987; Yi et al., 2001), PSYRATS-D and PSYRATS-Auditory Hallucinations (PSYRATS-AH) (Haddock, McCarron, Tarrier, & Faragher, 1999), Clinical Global Impression-Schizophrenia (CGI-SCH) (Busner & Targum, 2007), and Social and Occupational Functioning Assessment Scale (SOFAS) (Goldman, Skodol, & Lave, 1992). The raters were psychiatrists with at least 2 years of experience in administering these scales; they were blinded to the group assignments. The R-GPTS (Freeman et al., 2021), Brief Core Schema Scale (BCSS) (Fowler et al., 2006), Beck Depression Inventory (BDI) (Beck, Steer, & Brown, 1987), Brooding Scale (BS) (Kim et al., 2019), Fear of Negative Evaluation Scale (FNES) (Leary, 1983), Internal Personal and Situational Attribution Questionnaire (IPSAQ) (Kinderman & Bentall, 1996), and Self Esteem Scale (SES) (Rosenberg, 1965) self-rating scales also were used. Assessments were completed within the week before session 1 and within the week after session 10. The PSYRATS-D and R-GPTS scores were the primary outcome measures to operationalize DOR and persecutory IOR. VR sickness was assessed using the Simulator Sickness Questionnaire (Kennedy, Lane, Berbaum, & Lilienthal, 1993).

### Procedures

This study was a single-blind randomized clinical trial. Both the raters and the statistician were blinded to group assignments. During the trial, discussing patients by name or other potentially identifying information was strictly prohibited. The VR-T group completed 10 weekly sessions, each supervised by psychiatrists with at least 2 years of experience. Sessions began with a brief conversation (2–3 min) about the patients’ referential thinking over the past week. After donning the head-mounted display (HMD, Meta Quest 2), patients watched 5-min educational videos that were developed by the authors. There were three videos: the first, which addressed the concept and causes of IOR, was viewed during sessions 1–3; the second, focused on raising awareness of low self-esteem, negative thoughts, and past trauma related to IOR, was shown during sessions 4–7; and the third, which covered coping strategies to overcome IOR (including avoiding rumination, practicing self-assertion/healthy aggression, and reality testing), was presented during sessions 8–10. After patients had viewed the educational videos, they were exposed to 360° 3D three treatment videos depicting conversations among actors, all of whom were native Koreans, in settings such as a café, hospital, and walkway, or four animated videos set in a bus station, classroom, hospital, and walkway (Supplementary Figure 1). Patients were encouraged to select videos set in locations where they typically experienced IOR. The conversations either involved activities of daily life (‘chatting’) or focused on ‘gossiping’, with adjustable volume. Because patients were more sensitive to low-volume conversations and gossiping, the volume was controlled to facilitate reality testing. Depending on the duration of the video selected, two or three videos were played during each session which was adjusted to 20–25 min. Between the videos, psychiatrists asked patients whether, and to what extent, they experienced IOR, such as ‘Did you feel that they were talking about you?’ and ‘How confident are you and what percentage are you certain that they were definitely talking about you?’ The conviction percentage (0%–100%) was used in the next session to help patients recognize that the percentage can be decreased or changed when exposed to the same videos and learn that their thoughts could be flexible. For the purpose of reality testing, patients were encouraged not to feel intimidated, but rather to observe the actors’ faces and listen carefully so that they could be aware that the conversation was irrelevant to them.

The VR-C group was supervised by research nurses. These participants individually watched a single educational video across all 10 sessions and were instructed to relax while viewing subsequent control videos. The VR-C group watched the same seven videos as the VR-T group, except the actors’ voices were muted, or they watched 11 360° 3D videos of natural scenes. They selected the videos based on their preference but the average proportion of scenarios to natural scenes in total sessions was arranged to be 1 to 1. Typically, participants were exposed to two or three different control videos. Between the viewings, we simply asked them ‘How was it?’ or ‘Did you enjoy the scenes?’ Although inaudible conversations have elicited IOR in some patients, no therapeutic intervention was provided. The duration of a session and the number of total sessions were matched between the two groups. Both interventions were conducted in the digital therapy room in each hospital.

### Statistical analysis

Baseline variables were compared between the groups using the chi-square (χ^2^) test, Fisher’s exact test, or independent t-test; the paired t-test was used for within-group analyses. Intervention effects were analyzed using two-way repeated-measures (RM) analysis of variance. Two-way RM analysis of covariance was performed with the two variables (negative-self and negative-other schema from the BCSS) showing significant group differences included as covariates. Multiple testing of clinical scales was corrected for main effects and interaction using the False Discovery Rate (FDR). For post-hoc comparison, Bonferroni correction for two time points was applied. The correction was only applied to clinical scales because no significant results were observed with self-rating scales. Partial eta squared (η^2^p) was calculated for the estimation of group × time interaction effect size. According to Cohen, η^2^p value of ≥0.01 indicates a small effect, ≥ 0.06 represents a medium effect, and ≥0.14 is considered a large effect (Cohen, 2013). Additionally, Cohen d was calculated for the changes after intervention in each group. For the between- and within-group analyses of VR sickness, the χ^2^ test and McNemar test were conducted, respectively. All analyses were performed using SPSS for Windows (version 25.0; IBM Corp., Armonk, NY, USA).

Path analysis was conducted to evaluate causality in the relationships between dependent and independent variables. Psychological factors (attributional bias, depression, low self-esteem, negative beliefs, paranoia, rumination, and social-evaluative concern were measured by IPSAQ, BDI, SES, BCSS, R-GPTS, BS, and FNES, respectively.) potentially affecting the primary outcome measures were identified via correlation analysis and based on previous relevant studies. The IPSAQ and SES scores were excluded from the models due to a lack of correlation with the primary outcome measures (Supplementary Table 1). We selected the FNES, BDI, and BS scores as mediators based on previous studies (Cui et al., 2019; Fisher et al., 2013; Jaya, Ascone, & Lincoln, 2018; Lam et al., 2022; Morrison, Goldin, & Gross, 2024; Murphy, Murphy, & Shevlin, 2015). When the initial model did not show an adequate fit to the data, we removed nonsignificant paths to optimize the model. The model fit indices used were the χ^2^ p-value (cutoff, p > 0.05), comparative fit index (cutoff >0.95), root mean square error of approximation (cutoff <0.05), standardized root mean square residual (cutoff <0.09), and Tucker–Lewis index (cutoff >0.95) (West, Taylor, & Wu, 2012). The significance of indirect effects was confirmed using the bootstrap method with 2,000 iterations. If there were multiple indirect paths, phantom modeling was applied to calculate the indirect effect of a specific path. Mediators were classified as partial when both indirect and direct effects were significant and as full when only indirect effects were significant. Multiple testing was corrected using FDR.

## Results

### Participant characteristics

There were no significant differences in demographic or clinical characteristics between the two groups (Table 1). One and two individuals in VR-T and VR-C groups respectively were antipsychotic-free during the entire sessions. Six participants in the VR-T group and two participants in the control group dropped out for various reasons (Supplementary Figure 2).

**Table 1.** Demographic and clinical characteristics of the participants

Variables	VR-treatment (n = 32)	VR-control (n = 38)	Total (n = 70)	*p-*value
Sex				0.81
Male	17(53%)	22(58%)	39(56%)	
Female	15(47%)	16(42%)	31(44%)	
Age (years)	30.06 ± 8.65	29.97 ± 7.8	30.01 ± 8.12	0.96
Age of onset (years)	24.13 ± 8.06	21.21 ± 5.58	22.54 ± 6.93	0.08
Education (years)	13.85 ± 2.08	13.82 ± 1.94	13.83 ± 2.01	0.96
Diagnosis				0.57
SSDs^a^	24(75%)	31(82%)	55(79%)	
Other psychoses^b^	8(25%)	7(18%)	15(21%)	
Comorbid diagnoses	1(3.12%)	1(2.63%)	2(2.86%)	1.00
DI	85.22 ± 77.24	108.84 ± 88.88		0.24
Chlorpromazine equivalent dose (mg)				
Antipsychotic-free included	596.97 ± 419.51	606.33 ± 499.13	610.20 ± 473.18	0.93
Antipsychotic-free excluded	634.62 ± 79.24 (n = 31)	640.02 ± 491.11 (n = 36)	637.52 ± 455.18 (n = 67)	0.96

*Note:* Data are presented as mean ± standard deviation or %.

Abbreviations: DUP, Duration of Untreated Psychosis; DI, Duration of Illness; SSDs, Schizophrenia Spectrum Disorders; VR, Virtual Reality.

a SSDs refer to schizophrenia (n = 52), schizophreniform disorder (n = 2), schizoaffective disorder (n = 2).

b Other psychoses refers to other specified schizophrenia spectrum and other psychotic disorders (n = 13), and unspecified schizophrenia spectrum and other psychotic disorders (n = 1).

### Treatment effects

Within-group analysis showed improvements in the PANSS and PSYRATS-D scores in both groups; the PSYRATS-AH and CGI-SCH scores improved only in the VR-T group. Between-group analysis revealed a significant main effect of time and a significant group × time interaction at FDR-adjusted level for most measures. However, post hoc t-tests of the result after 10th session revealed significantly greater improvement in the PSYRATS-D score in the VR-T group compared with the VR-C group (emotional domain, Bonferroni-adjusted *p* = 0.02; total, Bonferroni-adjusted *p* = 0.04) (Table 2).

**Table 2.** Results of VR interventions on clinical scales^a^

Variables	VR-control			VR-treatment			Cohen’s d	Main effect				Group × Time interaction			*p^c^*	*p* ^d^
								Group		Time						
	Before 1^st^session	After 10^th^session	*p* ^b^	Before 1^st^session	After 10^th^session	*p* ^b^		F	*p*	F	*p*	F	*p*	η^2^p		
PANSS																
Positive symptoms	13.58 ± 3.79	12.97 ± 3.45	0.004	14.03 ± 3.78	11.97 ± 3.56	<0.001	−0.865	0.11	0.75	43.57	<0.001*	13.00	0.001*	0.160	0.62	0.24
Negative symptoms	12.55 ± 4.88	12.45 ± 4.80	0.50	12.25 ± 4.17	11.34 ± 3.62	0.02	−0.522	0.45	0.51	7.54	0.01*	4.73	0.03	0.065	0.78	0.29
General psychopathology	26.42 ± 5.49	25.32 ± 5.00	0.02	26.78 ± 4.69	23.91 ± 4.73	0.01	−0.569	0.21	0.65	28.48	<0.001*	5.63	0.02*	0.076	0.77	0.23
Total	52.55 ± 12.43	50.74 ± 11.57	0.01	53.06 ± 10.15	47.22 ± 9.70	0.002	−0.824	0.34	0.57	42.62	<0.001*	11.79	0.001*	0.148	0.85	0.18
PSYRATS-D																
Cognitive Factor	5.26 ± 4.32	4.76 ± 3.98	0.05	6.22 ± 3.78	2.78 ± 3.60	<0.001	−1.080	0.33	0.57	36.38	<0.001*	20.26	<0.001*	0.229	0.33	0.03
Emotional Factor	2.71 ± 2.58	2.26 ± 2.26	0.02	3.38 ± 2.32	1.03 ± 1.51	<0.001	−1.093	0.33	0.54	44.98	<0.001*	20.71	<0.001*	0.234	0.27	0.01^†^
Total	7.97 ± 6.75	7.03 ± 6.05	0.02	9.53 ± 5.84	3.81 ± 4.75	<0.001	−1.113	0.39	0.57	41.98	<0.001*	21.55	<0.001*	0.240	0.31	0.02^†^
PSYRATS-AH																
Cognitive Factor	2.29 ± 3.01	2.16 ± 2.65	0.41	2.00 ± 2.83	1.78 ± 2.64	0.05	−0.132	0.25	0.62	4.86	0.03*	0.30	0.59	0.004	0.68	0.55
Emotional Factor	3.47 ± 4.52	3.68 ± 4.43	0.29	3.50 ± 4.70	2.94 ± 4.48	0.06	−0.483	0.11	0.74	0.84	0.36	4.05	0.05	0.056	0.98	0.49
Physical Factor	3.16 ± 3.92	3.45 ± 4.01	0.51	3.31 ± 4.08	2.81 ± 3.70	0.01	−0.547	0.07	0.79	0.37	0.55	5.19	0.03	0.071	0.87	0.49
Total	8.92 ± 11.09	9.29 ± 10.81	1.00	8.81 ± 11.24	7.53 ± 10.34	0.01	−0.523	0.13	0.72	1.45	0.23	4.75	0.03*	0.065	0.97	0.49
CGI-SCH																
Positive Symptoms	3.00 ± 0.87	2.95 ± 0.90	0.26	3.13 ± 0.94	2.53 ± 1.02	0.001	−0.882	0.48	0.49	19.27	<0.001*	13.50	<0.001*	0.166	0.57	0.07
Negative Symptoms	2.53 ± 1.06	2.50 ± 0.95	0.16	2.59 ± 0.95	2.31 ± 0.90	0.03	−0.454	0.07	0.79	5.21	0.03*	3.58	0.06	0.050	0.78	0.40
Depressive Symptoms	2.82 ± 1.20	2.50 ± 1.16	0.89	2.84 ± 1.35	2.41 ± 1.34	0.02	−0.125	0.01	0.91	10.46	0.002*	0.27	0.60	0.004	0.93	0.75
Cognitive Symptoms	2.16 ± 1.00	2.24 ± 0.91	0.04	2.19 ± 0.64	2.16 ± 0.57	0.75	−0.229	0.02	0.89	0.17	0.68	0.91	0.34	0.013	0.89	0.67
Overall Severity	3.08 ± 0.75	3.00 ± 0.74	0.26	3.22 ± 0.87	2.78 ± 0.87	<0.001	−0.773	0.05	0.83	21.52	<0.001*	10.37	0.002*	0.132	0.47	0.26

Abbreviations: CGI-SCH, Clinical Global Impression-Schizophrenia; PSYRATS-AH, Psychotic Symptom Rating Scales-Auditory Hallucination; PSYRATS-D, Psychotic Symptom Rating Scales-Delusion; PANSS, Positive and Negative Syndrome Scale; SES, Self Esteem Scale; VR, Virtual reality.

a Two-way repeated measure ANOVA.

b Paired *t* test.

c Independent *t* test between treatment and control groups before 1st session.

d Independent *t* test between treatment and control groups after 10th session; Current p-value is uncorrected one;

* Significant (< 0.05) at false discovery rate correction (for each item, corrected 12 times and for total or overall severity, corrected four times);

† Significant after Bonferroni correction for two time points.

Regarding the self-rating measures, within-group analysis showed improvements in the BS and R-GPTS scores in the VR-C group and the SOFAS and R-GPTS scores in the VR-T group. Between-group analysis revealed a significant main effect of time for most measures, although the group × time interaction was significant only for the SOFAS score (p = 0.001). However, there were no significant differences in the SOFAS between groups in both results after 1st and 10th session (Table 3).

**Table 3.** Results of VR interventions on self-rating scales^a^

Variables	VR-control			VR-treatment			Cohen’s d	Main effect					Group Time interaction			*p* ^d^	*p* ^e^
								Group			Time						
	Before 1^st^session	After 10^th^session	*p* ^c^	Before 1^st^session	After 10^th^session	*p* ^c^		F	*p*		F	*p*	F	*p*	η^2^p		
SOFAS	59.95 ± 7.72	60.53 ± 7.84	0.08	58.53 ± 8.41	62.91 ± 8.35	<0.001	0.815	0.068	0.795		19.66	<0.001	11.54	0.001	0.145	0.47	0.22
BCSS																	
Negative-self^b^	10.76 ± 7.68	9.92 ± 8.27	0.06	7.47 ± 5.81	7.16 ± 7.14	0.69	0.169	3.32	0.07		1.816	0.18	0.50	0.48	0.007	0.05	0.58
Positive-self	9.18 ± 5.83	9.43 ± 5.77	0.11	10.41 ± 6.49	10.06 ± 6.33	0.67	−0.164	0.54	0.46		0.003	0.96	0.47	0.50	0.007	0.41	0.67
Negative-others^b^	9.13 ± 6.46	8.22 ± 6.59	0.59	6.06 ± 5.86	5.94 ± 5.80	0.86	0.200	3.78	0.06		1.142	0.29	0.70	0.41	0.010	0.04	0.93
Positive-others	8.47 ± 4.45	8.59 ± 5.37	0.21	9.63 ± 7.03	8.56 ± 6.48	0.39	−0.233	0.27	0.61		0.334	0.57	0.94	0.34	0.014	0.41	0.98
BDI	17.63 ± 13.58	15.54 ± 14.84	0.48	13.72 ± 11.09	11.72 ± 11.00	0.18	0.027	1.76	0.19		4.963	0.03	0.01	0.91	0.000	0.20	0.24
Brooding scale																	
Cognitive factor	9.1 ± 4.103	8.30 ± 4.37	0.07	7.66 ± 4.24	6.78 ± 4.46	0.15	−0.062	2.19	0.14		4.032	0.05	0.07	0.80	0.001	0.14	0.16
Emotional factor	10.53 ± 4.95	9.70 ± 5.14	0.03	8.19 ± 5.20	8.19 ± 4.74	1.00	0.237	2.86	0.10		1.264	0.27	1.26	0.27	0.019	0.06	0.21
Total	19.66 ± 8.59	18.00 ± 9.02	0.19	15.84 ± 9.03	14.97 ± 8.89	0.43	0.346	2.70	0.11		3.377	0.07	0.26	0.61	0.004	0.08	0.17
FNES	35.16 ± 13.73	32.86 ± 13.41	0.02	31.44 ± 13.91	31.09 ± 15.88	0.84	0.199	0.70	0.41		1.279	0.26	0.70	0.41	0.010	0.27	0.62
IPSAQ																	
Externalizing bias	0.95 ± 4.44	0.92 ± 4.15	0.15	1.50 ± 4.06	1.91 ± 15.88	0.63	0.123	0.61	0.44		0.088	0.77	0.26	0.60	0.004	0.59	0.37
Personalizing bias	0.38 ± 0.33	0.42 ± 0.35	0.85	0.41 ± 0.34	0.43 ± 0.39	0.95	−0.074	0.14	0.71		0.170	0.68	0.10	0.76	0.001	0.64	0.89
R-GPTS																	
Part 1: reference	15.68 ± 6.64	13.59 ± 7.97	0.32	15.44 ± 6.53	13.31 ± 7.32	0.05	0.007	0.04	0.85		9.844	0.003	0.001	0.98	0.000	0.88	0.88
Part 2: persecution	17.16 ± 10.49	15.14 ± 11.85	0.02	14.84 ± 9.16	12.34 ± 9.99	0.12	−0.039	1.26	0.27		6.057	0.02	0.03	0.87	0.000	0.33	0.30
Total	32.84 ± 16.04	28.73 ± 18.90	0.07	30.28 ± 13.78	25.66 ± 16.28	0.05	−0.022	0.63	0.43		9.376	0.003	0.01	0.93	0.000	0.48	0.48
SES	24.18 ± 7.36	24.51 ± 6.92	0.71	25.44 ± 5.89	25.63 ± 5.84	0.77	−0.072	0.67	0.42		0.510	0.48	0.09	0.77	0.001	0.44	0.48

Abbreviations: BCSS, Brief Core Schema Scale; BDI, Beck Depression Inventory; FNES, Fear of Negative Evaluation Scale; IPSAQ, Internal, Personal and Situational Attributions Questionnaire; R-GPTS, the Revised Green et al., Paranoid Thought Scale; SES, Self Esteem Scale; SOFAS, Social and Occupational Functioning Assessment Scale; VR, Virtual reality.

a Two-way repeated measure ANOVA.

b For the variables showed significant difference at baseline, two-way repeated measure ANCOVA was performed with the baseline data as covariates).

c Paired *t* test.

d Independent *t* test between treatment and control groups before 1^st^ session.

e Independent *t* test between treatment and control groups after 10^th^ session with the variables^b^ as covariates.

### VR sickness

The most common symptoms in both groups were general discomfort, fatigue/eye fatigue, difficulty focusing the eyes, and difficulty concentrating. Within-group analyses did not show significant changes in either group. Significant group differences were observed in general discomfort (p = 0.05), sweating (p = 0.05), and difficulty concentrating (p = 0.03), but only after session 1 (Table 4). There were no significant group differences in the severity of VR sickness symptoms after sessions 1 and 10, except for blurry vision (p = 0.02) (Supplementary Table 2).

**Table 4.** Symptoms of VR sickness reported after 1^st^ and after 10^th^ session

Symptoms	VR-control			VR-treatment				Between-group comparison	
	After 1^st^ session (n = 38)	After 10^th^ session (n = 37)	*p* ^a^	After 1^st^ session (n = 32)		After 10^th^ session (n = 32)	*p* ^a^	*p^b^after* *1*^st^ *session*	*p* ^b^after10^th^ session
General discomfort	21 (55.26%)	22 (59.46%)	1.00	1.00	25 (78.13%)	19 (59.38%)	0.15	0.05	0.99
Fatigue	19 (50.00%)	22 (59.46%)	0.29	0.29	20 (62.50%)	21 (65.63%)	1.00	0.29	0.60
Headache	11 (28.95%)	13 (35.14%)	0.75	0.75	10 (31.25%)	11 (34.38%)	1.00	0.83	0.95
Eye fatigue	20 (52.63%)	24 (64.86%)	0.30	0.30	20 (62.50%)	17 (53.13%)	0.58	0.41	0.32
Difficulty focusing the eyes	24 (63.16%)	18 (48.65%)	0.30	0.30	20 (62.50%)	12 (37.50%)	0.06	0.96	0.35
Increased salivation	6 (15.79%)	8 (21.62%)	0.69	0.69	6 (18.75%)	7 (21.88%)	1.00	0.74	0.98
Sweating	3 (7.89%)	6 (16.22%)	0.38	0.38	8 (25.00%)	3 (9.38%)	0.13	0.05	0.40
Nausea	11 (28.95%)	13 (35.14%)	0.77	0.77	8 (25.00%)	11 (34.38%)	0.51	0.71	0.95
Difficulty in concentrating	15 (39.47%)	15 (40.54%)	1.00	1.00	21 (65.63%)	17 (53.13%)	0.29	0.03	0.30
Head feels full	10 (26.32%)	15 (40.54%)	0.27	0.27	15 (46.88%)	13 (40.63%)	0.73	0.07	0.99
Blurry vision	19 (50.00%)	13 (35.14%)	0.30	0.30	16 (50.00%)	11 (34.38%)	0.23	1.00	0.95
Dizziness when opening eyes	13 (34.21%)	12 (32.43%)	1.00	1.00	10 (31.25%)	7 (21.88%)	0.45	0.79	0.33
Dizziness when eyes are closed	9 (23.68%)	8 (21.62%)	1.00	1.00	10 (31.25%)	9 (28.13%)	1.00	0.48	0.53
Dizziness with a spinning feeling	15 (39.47%)	14 (37.84%)	1.00	1.00	15 (46.88%)	13 (40.63%)	0.69	0.53	0.81
Feeling burdened by the stomach	8 (21.05%)	13 (35.14%)	0.23	0.23	6 (18.75%)	8 (25.00%)	0.69	0.81	0.36
Burp	4 (10.53%)	7 (18.92%)	0.45	0.45	3 (9.38%)	8 (25.00%)	0.13	0.87	0.54

Abbreviation: VR, Virtual reality.

a McNemar test for comparing the proportion of patients complaining VR sickness within group between two time points.

b Chi-square test for comparing two groups.

### Path analysis

**Figure 1.**
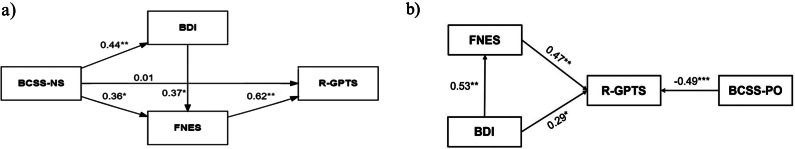
Path models between the BCSS-NS or BCSS-PO and R-GPTS: (a) Model depicting paths between the BCSS-NS and R-GPTS (Fit indices: chi-square = 0.97; D.*F* = 1; CFI = 1; TLI = 1.01; RMSEA = 0; SRMR = 0.04; *p* = 0.97) and (b) Model depicting paths between the BCSS-PO and R-GPTS. Fit indices: chi-square = 0.85; D.*F* = 1; CFI = 1; TLI = 1.02; RMSEA = 0; SRMR = 0.05; *p* = 0.36; For FDR-adjusted level, * *p <* .05, ** *p* < .01, ***p < .001. **Note**. BCSS-NS, Brief Core Schema Scale-Negative Self; BCSS-PO, Brief Core Schema Scale-Positive Others; BDI, Beck Depression Inventory; FNES, Fear of Negative Evaluation Scale; R-GPTS, the Revised Green et al., Paranoid Thought Scale.

When running the analysis between the BCSS-NS and R-GPTS (total score) in the VR-T group, an optimal model showed significant direct effects between the FNES and R-GPTS (r = 0.62, FDR-adjusted *p* < 0.0005), the BCSS-NS and BDI (r = 0.44, FDR-adjusted *p* = 0.0025), the BDI and FNES, and the BCSS-NS and FNES (r = 0.37, FDR-adjusted *p* = 0.0167; r = 0.36, FDR-adjusted *p* = 0.0263, respectively). There were significant indirect effects between the BCSS-NS and R-GPTS (r = 0.33, FDR-adjusted *p* = 0.018) and between the BCSS-NS and FNES (r = 0.162, FDR-adjusted *p* = 0.0285). When the former path was broken down into two paths (BCSS-NS → BDI → FNES → R-GPTS and BCSS-NS → FNES → R-GPTS) using phantom modeling, only FNES in the second path was a significant full mediator (r = 0.224, uncorrected *p* = 0.04). For the latter path, BDI played a partial mediating role. The analysis between the BCSS-PO and R-GPTS showed significant direct effects between the BCSS-PO and R-GPTS (r = −0.49, FDR-adjusted *p* < 0.0002), the BDI and R-GPTS (r = 0.29, FDR-adjusted *p* = 0.024), and the FNES and R-GPTS (r = 0.47, FDR-adjusted *p* = 0.0067) and between the BDI and FNES (r = 0.53, FDR-adjusted *p* < 0.0002). A significant indirect effect was identified only between the BDI and R-GPTS (r = 0.25, uncorrected *p* = 0.03). In this path, FNES played a partial mediating role (Figure 1). On the other hand, analysis between the BCSS-NS and PSYRATS-D (total score) showed no significant results.

## Discussion

IOR and DOR are common symptoms of psychosis. Recently, interest in VR therapy for individuals with psychosis has been increasing. We investigated the effects and safety of VR-T in relation to persecutory IOR and DOR in stable outpatients with psychosis. After session 10, a large and significant effect size was observed especially for the PSYRATS-D score. Although VR sickness had a high incidence, its symptoms generally were mild and transient.

Within-group analysis revealed significant improvements in PANSS, PSYRATS-D, and CGI-SCH scores were observed in both groups, although PSYRATS-AH scores improved only in the VR-T group. Importantly, between-group analysis revealed significant group × time interactions in most of the clinical scales. However, in the post hoc test, a significant difference was only observed in the PSYRATS-D scores after the 10th session. For the results other than the PSYRATS-D, it may signify that the effects of VR therapy become evident over time but not enough to produce significant differences between the two groups after the 10th session. Or it may be that a significant difference was noted between the 1st and 10th sessions. If so, measurement at a minimum of three time points (baseline, mid-point, and end-point) should be considered in future studies. The result of the PSYRATS-D, suggests that VR therapy is beneficial in reducing the severity of delusions among stable outpatients with psychosis. The effect size (Cohen d) was larger than that reported in a meta-analysis of cognitive behavioral therapy (CBT) effects on delusions (0.36) (Van der Gaag, Valmaggia, & Smit, 2014). Because the primary outcome measures in previous VR studies differed from those in our study, direct comparisons of the results were difficult. In one study (Freeman et al., 2016), the baseline mean PSYRATS-D score was ~17, which is higher than the score in our study. This suggests that VR therapy is a promising tool to reduce persecutory IOR and DOR, even in patients with mild symptoms. The negative findings regarding the PANSS scores in the post hoc test may be related to the mildness of the patients’ symptoms and the fact that the PANSS is not sufficiently sensitive to detect subtle changes in symptoms.

For self-rating scales, within-group comparisons showed some improvement in the BS and R-GPTS scores in both groups, but there were no significant differences between the groups. Although these findings might appear disappointing, they may reflect the differences between clinical and self-rating scales. Thus, the effects of the VR-T and VR-C interventions on functioning, schema, depression, brooding, negative evaluation, attribution bias, paranoia, and self-esteem subscale scores were minimal. It should be noted that Freeman et al. (2022) reported no significant change in R-GPTS scores after six sessions of VR therapy, and the baseline R-GPTS scores were low in a manner similar to the results in our study. However, Pot-Kolder et al. (2018) reported significant changes in R-GPTS scores after 16 sessions of VR-CBT, although the baseline R-GPTS scores were very high. Hence, it seems that negative findings in the R-GPTS may be related to the low baseline score or a floor effect and limited number of sessions.

Regarding safety, both groups had a moderate-to-high incidence of VR sickness. The most common symptoms in the VR-T group were general discomfort, fatigue, and difficulty concentrating; in the control group, difficulty focusing the eyes and eye fatigue were more prevalent. However, the symptoms generally were mild, and there were no significant group differences in symptom frequency. These findings indicate that our VR intervention is safe for patients with psychosis, consistent with other studies (Fornells-Ambrojo et al., 2008; Rus-Calafell et al., 2018). This safety may be reflected in the low attrition rates observed in both the VR-T and VR-C groups (15.8% and 5.0%, respectively), which could be considered a strength of VR therapy, especially in young individuals with psychosis. However, it is of note that six participants were dropped out in the VT-T group whereas only two in the VR-C group. Unfortunately, in the VR-T group, one completed suicide and one aggravated due to self-discontinuation of medication. For the former case, we were unable to evaluate association with the VR exposure but for the latter case, no association was found. In general, suicide or symptom aggravation due to VR exposure in psychosis is scarce. Two recent VR studies in SSD reported suicide attempts in the treatment group but its rate was not different compared with the control group (Glenthøj et al., 2024a, 2024b). Nevertheless, these adverse events should be carefully monitored in future studies.

In our path model of the BCSS-NS and R-GPTS scores, the FNES score acted as a full mediator in the VR-T group. This indicates that the BCSS-NS score did not directly affect the R-GPTS score; its influence was indirectly mediated through the FNES score. Additionally, the BDI score served as a partial mediator in the relationship between the BCSS-NS and FNES scores. These findings suggest that psychological and VR therapies targeting paranoia should focus on negative evaluations and depression. We found that the BCSS-PO score was independently and negatively associated with the R-GPTS score, and the FNES score was a partial mediator in the relationship between the BDI and R-GPTS scores. Taken together, the results of our path analyses provide insights into how paranoia may be improved. There was no mediating effect of the BS score on the relationship between the BCSS-NS and PSYRATS-D scores.

Several limitations of the current study should be noted. First is related to the appropriateness of VR-C which consisted of the same videos used in the VR-T group but with muted voices or 360°-3D videos of nature scenes. We chose these conditions to test the efficacy of IOR-related contents versus neutral ones. Nevertheless, as some short reality testing approach was applied in the VR-T, offline CBT sessions could be an alternative control. In addition, mismatching the number of educational videos between the groups might have introduced some bias. Second, because the participants were young and stable outpatients, the generalizability of the findings is limited. Third, the actors in the videos generally were young, and some middle-aged participants felt that the scenarios were not adequately relatable. Fourth, as we evaluated outcomes only at two time points, we may have overlooked significant changes that occurred in the middle. Additionally, one participant dropped out because the HMD did not fit their head size. These issues should be addressed in future studies. As a strength of the study, this was the first VR intervention targeting IOR and DOR in Asian patients with psychosis. Moreover, the scenarios and volume could be adjusted to facilitate reality testing.

In conclusion, our findings suggest that VR therapy is beneficial in reducing delusions in stable young outpatients with psychosis; symptoms of VR sickness were mild and tolerable. Future studies should develop more diverse videos to better meet the needs of older participants.

## Supporting information

Jeon et al. supplementary material Jeon et al. supplementary material

## Supplementary material

To view supplementary material for this article, please visit http://doi.org/10.1017/S0033291725000959.

## References

[r1] American Psychiatric Association. (2013). Diagnostic and statistical manual of mental disorders (5th ed.). APA. 10.1176/appi.books.9780890425596

[r2] Beck, A. T., Steer, R. A., & Brown, G. K. (1987). Beck depression inventory. San Antonio, TX: Psychological Corporation

[r3] Bendala-Rodríguez, P., Senín-Calderón, C., Peluso-Crespi, L., & Rodríguez-Testal, J. F. (2019). Vulnerability to psychosis, ideas of reference and evaluation with an implicit test. Journal of Clinical Medicine, 8(11), 1956. 10.3390/jcm8111956 31766179 PMC6912563

[r4] Busner, J., & Targum, S. D. (2007). The clinical global impressions scale: Applying a research tool in clinical practice. Psychiatry (Edgmont), 4(7), 28. PMC2880930 20526405

[r5] Chan, C. L., Ngai, E. K., Leung, P. K., & Wong, S. (2010). Effect of the adapted virtual reality cognitive training program among Chinese older adults with chronic schizophrenia: A pilot study. International Journal of Geriatric Psychiatry: A Journal of the Psychiatry of Late Life and Allied Sciences, 25(6), 643–649. 10.1002/gps.2403 19806599

[r6] Cicero, D. C., & Kerns, J. G. (2011). Unpleasant and pleasant referential thinking: Relations with self-processing, paranoia, and other schizotypal traits. Journal of Research in Personality, 45(2), 208–218. 10.1016/j.jrp.2011.02.002 26028792 PMC4447705

[r7] Cohen, J. (2013). Statistical power analysis for the behavioral sciences. Routledge.

[r8] Colori, S. (2017). Understanding referential thinking. Schizophrenia Bulletin, 43(4), 685–686. 10.1093/schbul/sbv005 25681762 PMC5472136

[r9] Cui, Y., Kim, S. W., Lee, B. J., Kim, J. J., Yu, J. C., Lee, K. Y., … & Chung, Y. C. (2019). Negative schema and rumination as mediators of the relationship between childhood trauma and recent suicidal ideation in patients with early psychosis. The Journal of Clinical Psychiatry, 80(3), 11438. 10.4088/JCP.17m12088 30946541

[r10] Dellazizzo, L., Potvin, S., Phraxayavong, K., & Dumais, A. (2021). One-year randomized trial comparing virtual reality-assisted therapy to cognitive–behavioral therapy for patients with treatment-resistant schizophrenia. NPJ Schizophrenia, 7(1), 9. 10.1038/s41537-021-00139-2 33580033 PMC7881089

[r11] Fajnerová, I., Rodriguez, M., Levčík, D., Konrádová, L., Mikoláš, P., Brom, C., … & Horáček, J. (2014). A virtual reality task based on animal research–spatial learning and memory in patients after the first episode of schizophrenia. Frontiers in Behavioral Neuroscience, 8, 157. 10.3389/fnbeh.2014.00157 24904329 PMC4034703

[r12] Fisher, H. L., Schreier, A., Zammit, S., Maughan, B., Munafò, M. R., Lewis, G., & Wolke, D. (2013). Pathways between childhood victimization and psychosis-like symptoms in the ALSPAC birth cohort. Schizophrenia Bulletin, 39(5), 1045–1055. 10.1093/schbul/sbs088 22941743 PMC3756772

[r13] Fornells-Ambrojo, M., Barker, C., Swapp, D., Slater, M., Antley, A., & Freeman, D. (2008). Virtual reality and persecutory delusions: Safety and feasibility. Schizophrenia Research, 104(1–3), 228–236. 10.1016/j.schres.2008.05.013 18571899

[r14] Fowler, D., Freeman, D., Smith, B., Kuipers, E., Bebbington, P., Bashforth, H., … & Dunn, G. (2006). The Brief Core Schema Scales (BCSS): Psychometric properties and associations with paranoia and grandiosity in non-clinical and psychosis samples. Psychological Medicine, 36(6), 749–759. https://doi.org/10.1017/S0033291719003155ranges, and clinical cut-offs. Psychological Medicine, 51(2), 244–253. 10.1017/S0033291719003155 16563204

[r63] Freeman, D., Garety, P. A., Kuipers, E., Fowler, D., & Bebbington, P. E. (2002). A cognitive model of persecutory delusions. British Journal of Clinical Psychology, 41(4), 331–347. 10.1348/014466502760387461 12437789

[r61] Freeman, D., Garety, P. A., Bebbington, P. E., Smith, B., Rollinson, R., Fowler, D., … Dunn, G. (2005). Psychological investigation of the structure of paranoia in a non-clinical population. British Journal of Psychiatry, 186(5), 427–435. doi:10.1192/bjp.186.5.427 15863749

[r60] Freeman, D., Bradley, J., Antley, A., Bourke, E., DeWeever, N., Evans, N., … Dunn, G. (2016). Virtual reality in the treatment of persecutory delusions: randomised controlled experimental study testing how to reduce delusional conviction. The British Journal of Psychiatry, 209(1), 62–67. 10.1192/bjp.bp.115.176438 27151071 PMC4929408

[r15] Freeman, D., Reeve, S., Robinson, A., Ehlers, A., Clark, D., Spanlang, B., & Slater, M. (2017). Virtual reality in the assessment, understanding, and treatment of mental health disorders. Psychological Medicine, 47(14), 2393–2400. 10.1017/S003329171700040X 28325167 PMC5964457

[r65] Freeman, D., Loe, B. S., Kingdon, D., Startup, H., Molodynski, A., Rosebrock , … Bird, J. C. (2021). The revised Green et al., Paranoid Thoughts Scale (R-GPTS): psychometric properties, severity ranges, and clinical cut-offs. Psychological medicine, 51(2), 244–253. 10.1017/S0033291719003155 31744588 PMC7893506

[r64] Freeman, D., Lambe, S., Kabir, T., Petit, A., Rosebrock, L., Yu, L.-M., … O’Regan, E. (2022). Automated virtual reality therapy to treat agoraphobic avoidance and distress in patients with psychosis (gameChange): a multicentre, parallel-group, single-blind, randomised, controlled trial in England with mediation and moderation analyses. The Lancet Psychiatry, 9(5), 375–388. 35395204 10.1016/S2215-0366(22)00060-8 PMC9010306

[r16] Gega, L., White, R., Clarke, T., Turner, R., & Fowler, D. (2013). Virtual environments using video capture for social phobia with psychosis. Cyberpsychology, Behavior, and Social Networking, 16(6), 473–479. 10.1089/cyber.2013.1510 23659722 PMC3678564

[r17] Geraets, C. N., Snippe, E., van Beilen, M., Pot-Kolder, R. M., Wichers, M., van der Gaag, M., & Veling, W. (2020). Virtual reality based cognitive behavioral therapy for paranoia: Effects on mental states and the dynamics among them. Schizophrenia Research, 222, 227–234. 10.1016/j.schres.2020.05.047 32527676

[r18] Glenthøj, L., Smith, L., Vernal, D., Mariegaard, L., Christensen, A., Jansen, J., … Nordentoft, M. (2024a). Virtual reality-assisted therapy targeting persistent auditory verbal hallucinations in patients diagnosed with schizophrenia spectrum disorders: The Challenge single-blind, randomized clinical trial. Research Square. 10.21203/rs.3.rs-5180922/v1

[r19] Glenthøj, L., Vernal, D., Due, A. S., Mariegaard, L., Pinkham, A., Austin, S., … Jeppesen, U. (2024b). Face Your Fears: Virtual reality-based cognitive behavioral therapy (VR-CBT) versus standard CBT for paranoia in patients with schizophrenia spectrum disorders: Results of a randomized clinical trial. Research Square. 10.21203/rs.3.rs-5593252/v1

[r20] Goldman, H. H., Skodol, A. E., & Lave, T. R. (1992). Revising axis V for DSM-IV: A review of measures of social functioning. The American Journal of Psychiatry, 149, 9. 1386964 10.1176/ajp.149.9.1148

[r21] Haddock, G., McCarron, J., Tarrier, N., & Faragher, E. (1999). Scales to measure dimensions of hallucinations and delusions: The psychotic symptom rating scales (PSYRATS). Psychological Medicine, 29(4), 879–889. 10.1017/S0033291799008661 10473315

[r22] Jaya, E. S., Ascone, L., & Lincoln, T. (2018). A longitudinal mediation analysis of the effect of negative-self-schemas on positive symptoms via negative affect. Psychological Medicine, 48(8), 1299–1307. 10.1017/S003329171700277X 28956520

[r23] Kaney, S., & Bentall, R. P. (1989). Persecutory delusions and attributional style. British Journal of Medical Psychology, 62(2), 191–198. 10.1111/j.2044-8341.1989.tb02826.x 2751948

[r24] Kay, S. R., Fiszbein, A., & Opler, L. A. (1987). The positive and negative syndrome scale (PANSS) for schizophrenia. Schizophrenia Bulletin, 13(2), 261–276. 10.1093/schbul/13.2.261 3616518

[r25] Kennedy, R. S., Lane, N. E., Berbaum, K. S., & Lilienthal, M. G. (1993). Simulator sickness questionnaire: An enhanced method for quantifying simulator sickness. The International Journal of Aviation Psychology, 3(3), 203–220. 10.1207/s15327108ijap0303_3

[r26] Kim, J. H., Piao, Y., Kim, W. S., Park, J. J., Kang, N. I., Lee, K. H., & Chung, Y. C. (2019). The development of the brooding scale. Psychiatry Investigation, 16(6), 443. 10.30773/pi.2019.04.16 31247703 PMC6603696

[r27] Kinderman, P., & Bentall, R. P. (1996). A new measure of causal locus: The internal, personal and situational attributions questionnaire. Personality and Individual Differences, 20(2), 261–264. 10.1016/0191-8869(95)00186-7

[r28] Ku, J., Han, K., Lee, H. R., Jang, H. J., Kim, K. U., Park, S. H., … Kim, S. I. (2007). VR-based conversation training program for patients with schizophrenia: A preliminary clinical trial. Cyberpsychology & Behavior, 10(4), 567–574. 10.1089/cpb.2007.9989 17711366

[r29] Lam, A. H. Y., Cheung, Y. T. D., Wong, K. H., Leung, S. F., & Chien, W. T. (2022). Dispositional mindfulness and psychotic symptoms in schizophrenia spectrum disorders: The mediating roles of rumination and negative emotion. Neuropsychiatric Disease and Treatment, 18, 75. 10.2147/NDT.S338133 35046658 PMC8760986

[r30] Leary, M. R. (1983). A brief version of the fear of negative evaluation scale. Personality and Social Psychology Bulletin, 9(3), 371–375. 10.1177/0146167283093007

[r31] Lee, B. M., Kim, S.-W., Lee, B. J., Won, S.-H., Park, Y.-H., Kang, C. Y., … Chung, Y.-C. (2023). Effects and safety of virtual reality-based mindfulness in patients with psychosis: A randomized controlled pilot study. Schizophrenia, 9(1), 57. 10.1038/s41537-023-00391-8 37704650 PMC10499950

[r32] Leucht, S., Samara, M., Heres, S., & Davis, J. M. (2016). Dose equivalents for antipsychotic drugs: The DDD method. Schizophrenia Bulletin, 42(suppl_1), S90– S94. 10.1093/schbul/sbv167 27460622 PMC4960429

[r33] Lyon, H. M., Kaney, S., & Bentall, R. P. (1994). The defensive function of persecutory delusions: Evidence from attribution tasks. The British Journal of Psychiatry, 164(5), 637–646. 10.1192/bjp.164.5.637 7921714

[r34] Martens, M. A., Antley, A., Freeman, D., Slater, M., Harrison, P. J., & Tunbridge, E. M. (2019). It feels real: Physiological responses to a stressful virtual reality environment and its impact on working memory. Journal of Psychopharmacology, 33(10), 1264–1273. 10.1177/02698811198601 31294651 PMC6764008

[r35] Meyer, E. C., & Lenzenweger, M. F. (2009). The specificity of referential thinking: A comparison of schizotypy and social anxiety. Psychiatry Research, 165(1–2), 78–87. 10.1016/j.psychres.2007.10.015 19059651

[r36] Moritz, S., Voigt, M., Köther, U., Leighton, L., Kjahili, B., Babur, Z., …& Grzella, K. (2014). Can virtual reality reduce reality distortion? Impact of performance feedback on symptom change in schizophrenia patients. Journal of Behavior Therapy and Experimental Psychiatry, 45(2), 267–271. 10.1016/j.jbtep.2013.11.005 24384509

[r37] Morrison, A. S., Goldin, P. R., & Gross, J. J. (2024). Fear of negative and positive evaluation as mediators and moderators of treatment outcome in social anxiety disorder. Journal of Anxiety Disorders, 104, 102874. 10.1016/j.janxdis.2024.102874 38754336

[r38] Murphy, S., Murphy, J., & Shevlin, M. (2015). Negative evaluations of self and others, and peer victimization as mediators of the relationship between childhood adversity and psychotic experiences in adolescence: The moderating role of loneliness. British Journal of Clinical Psychology, 54(3), 326–344. 10.1111/bjc.12077 25756545

[r39] Park, K. M., Ku, J., Choi, S. H., Jang, H. J., Park, J. Y., Kim, S. I., & Kim, J. J. (2011). A virtual reality application in role-plays of social skills training for schizophrenia: A randomized, controlled trial. Psychiatry Research, 189(2), 166–172. 10.1016/j.psychres.2011.04.003 21529970

[r40] Pot-Kolder, R. M., Geraets, C. N., Veling, W., van Beilen, M., Staring, A. B., Gijsman, H. J., … & van der Gaag, M. (2018). Virtual-reality-based cognitive behavioural therapy versus waiting list control for paranoid ideation and social avoidance in patients with psychotic disorders: A single-blind randomised controlled trial. The Lancet Psychiatry, 5(3), 217–226. 29429948 10.1016/S2215-0366(18)30053-1

[r41] Rodríguez-Testal, J. F., Bendala-Rodríguez, P., Perona-Garcelán, S., & Senín-Calderón, C. (2019). Examining the structure of ideas of reference in clinical and community samples. Comprehensive Psychiatry, 93, 48–55. 10.1016/j.comppsych.2019.06.006 31340191

[r42] Rosenberg, M. (1965). Rosenberg self-esteem scale (RSE). Acceptance and commitment therapy. Measures package. APA PsycTests, 61(52), 18.

[r43] Rus-Calafell, M., Garety, P., Sason, E., Craig, T. J., & Valmaggia, L. R. (2018). Virtual reality in the assessment and treatment of psychosis: A systematic review of its utility, acceptability and effectiveness. Psychological Medicine, 48(3), 362–391. 10.1017/S0033291717001945 28735593

[r44] Ruse, S. A., Harvey, P. D., Davis, V. G., Atkins, A. S., Fox, K. H., & Keefe, R. S. (2014). Virtual reality functional capacity assessment in schizophrenia: Preliminary data regarding feasibility and correlations with cognitive and functional capacity performance. Schizophrenia Research: Cognition, 1(1), e21–e26. 10.1016/j.scog.2014.01.004 25083416 PMC4113005

[r45] Schennach, R., Riedel, M., Obermeier, M., Spellmann, I., Musil, R., Jäger, M., …& Naber, D. (2015). What are residual symptoms in schizophrenia spectrum disorder? Clinical description and 1-year persistence within a naturalistic trial. European Archives of Psychiatry and Clinical Neuroscience, 265, 107–116. 10.1007/s00406-014-0528-2 25261210

[r46] Tsang, M. M., & Man, D. W. (2013). A virtual reality-based vocational training system (VRVTS) for people with schizophrenia in vocational rehabilitation. Schizophrenia Research, 144(1–3), 51–62. 10.1016/j.schres.2012.12.024 23356951

[r47] Valmaggia, L. R., Freeman, D., Green, C., Garety, P., Swapp, D., Antley, A., … McGuire, P. K. (2007). Virtual reality and paranoid ideations in people with an ‘at-risk mental state’ for psychosis. British Journal of Psychiatry, 191(S51), s63–s68. 10.1192/bjp.191.51.s63 18055940

[r48] Van der Gaag, M., Valmaggia, L. R., & Smit, F. (2014). The effects of individually tailored formulation-based cognitive behavioural therapy in auditory hallucinations and delusions: A meta-analysis. Schizophrenia Research, 156(1), 30–37. 10.1016/j.schres.2014.03.016 24731619

[r49] Veling, W., Pot-Kolder, R., Counotte, J., van Os, J., & van der Gaag, M. (2016). Environmental social stress, paranoia and psychosis liability: A virtual reality study. Schizophrenia Bulletin, 42(6), 1363–1371. 10.1093/schbul/sbw031 27038469 PMC5049523

[r50] West, S. G., Taylor, A. B., & Wu, W. (2012). Model fit and model selection in structural equation modeling. Handbook of Structural Equation Modeling, 1(1), 209–231.

[r51] Wong, S. M., Hui, C. L., Wong, C. S., Suen, Y., Chan, S. K., Lee, E. H., Chang, W., … & Chen, E. Y. (2021). Induced ideas of reference during social unrest and pandemic in Hong Kong. Schizophrenia Research, 229, 46–52. 10.1016/j.schres.2021.01.027 33618286

[r52] World Health Organization. (1973). Report of the international pilot study of schizophrenia (Vol. 1). World Health Organization.

[r53] Yi, J. S., Ahn, Y. M., Shin, H. K., An, S. K., Joo, Y. H., Kim, S. H., … & Kim, Y. S. (2001). Reliability and validity of the Korean version of the positive and negative syndrome scale. Journal of Korean Neuropsychiatric Association, 40, 1090–1105.

