# Supplementary material for: Virtual reality therapy targeting ideas of reference in patients with psychosis: a single-blind parallel-group randomized controlled trial
Source: Psychol Med. 2025 Apr 28;55:e121. doi: 10.1017/S0033291725000959 (PMC12115268; doi:10.1017/S0033291725000959)
Supplement: Jeon et al. supplementary material [file S0033291725000959sup001.docx]

Suppl Table 1. Correlation results among the variables.

|  | 1 | 2 | 3 | 4 | 5 | 6 | 7 | 8 | 9 | 10 | 11 | 12 | 13 | 14 | 15 | 16 | 17 | 18 | 19 |
| --- | --- | --- | --- | --- | --- | --- | --- | --- | --- | --- | --- | --- | --- | --- | --- | --- | --- | --- | --- |
| 1 (PsyD-tot) | 1 |  |  |  |  |  |  |  |  |  |  |  |  |  |  |  |  |  |  |
| 2 (PsyD-cog) | 0.97*** | 1 |  |  |  |  |  |  |  |  |  |  |  |  |  |  |  |  |  |
| 3 (PsyD-emo) | 0.94*** | 0.83*** | 1 |  |  |  |  |  |  |  |  |  |  |  |  |  |  |  |  |
| 4 (R-GPTS-tot) | -0.06 | -0.16 | 0.02 | 1 |  |  |  |  |  |  |  |  |  |  |  |  |  |  |  |
| 5 (R-GPTS 1) | -0.16 | -0.24 | -0.11 | 0.85*** | 1 |  |  |  |  |  |  |  |  |  |  |  |  |  |  |
| 6 (R-GPTS 2) | 0.02 | -0.07 | 0.12 | 0.92*** | 0.59*** | 1 |  |  |  |  |  |  |  |  |  |  |  |  |  |
| 7 (EB) | -0.02 | 0.01 | -0.03 | -0.22 | -0.24 | -0.17 | 1 |  |  |  |  |  |  |  |  |  |  |  |  |
| 8 (PB) | 0.13 | 0.08 | 0.13 | 0.35 | 0.28 | 0.34 | -0.24 | 1 |  |  |  |  |  |  |  |  |  |  |  |
| 9 (PANSS-p) | 0.41* | 0.41* | 0.39* | -0.07 | 0.01 | -0.11 | -0.25 | 0.14 | 1 |  |  |  |  |  |  |  |  |  |  |
| 10 (BS-tot) | -0.41* | -0.42* | -0.33 | 0.24 | 0.22 | 0.21 | -0.11 | 0.17 | -0.16 | 1 |  |  |  |  |  |  |  |  |  |
| 11 (BS-emo) | -0.19 | -0.19 | -0.15 | 0.27 | 0.15 | 0.31 | -0.1 | 0.22 | 0.05 | 0.89*** | 1 |  |  |  |  |  |  |  |  |
| 12 (BS-cog) | -0.53** | -0.56*** | -0.44* | 0.15 | 0.23 | 0.07 | -0.09 | 0.08 | -0.35 | 0.88*** | 0.57*** | 1 |  |  |  |  |  |  |  |
| 13 (BCSS-NS) | -0.48** | -0.44* | -0.48** | 0.38* | 0.31 | 0.36* | -0.18 | -0.11 | -0.23 | 0.22 | 0.23 | 0.17 | 1 |  |  |  |  |  |  |
| 14 (BCSS-PS) | 0.09 | 0.04 | 0.16 | -0.04 | -0.11 | 0.02 | 0.28 | -0.09 | -0.01 | -0.05 | 0.01 | -0.1 | -0.35 | 1 |  |  |  |  |  |
| 15 (BCSS-NO) | -0.16 | -0.17 | -0.14 | -0.02 | 0.03 | -0.05 | 0.33 | -0.04 | -0.2 | 0.4* | 0.43* | 0.28 | 0.25 | 0.13 | 1 |  |  |  |  |
| 16 (BCSS-PO) | -0.22 | -0.15 | -0.27 | -0.44* | -0.22 | -0.52** | 0.16 | -0.24 | -0.01 | -0.24 | -0.18 | -0.24 | -0.04 | 0.27 | 0.06 | 1 |  |  |  |
| 17 (SES) | 0.04 | 0.01 | 0.09 | -0.35 | -0.33 | -0.29 | 0.49** | -0.31 | -0.08 | -0.11 | -0.14 | -0.06 | -0.28 | 0.68*** | 0.4* | 0.3 | 1 |  |  |
| 18 (BDI) | -0.08 | -0.12 | -0.04 | 0.45* | 0.48** | 0.34 | 0.07 | 0.02 | 0.02 | -0.04 | 0.04 | -0.12 | 0.45* | -0.12 | 0.05 | 0.17 | -0.31 | 1 |  |
| 19 (FNES) | -0.23 | -0.27 | -0.16 | 0.72*** | 0.72*** | 0.59*** | -0.16 | 0.11 | -0.1 | 0.33 | 0.4* | 0.2 | 0.51** | -0.12 | 0.1 | -0.07 | -0.37* | 0.55** | 1 |

*p* ≥ 0.05, **p*<.05. ***p*<.01. ****p*<.001.

*Note.* BCSS-NO, Brief Core Schema Scale-Negative Others; BCSS-NS, Brief Core Schema Scale-Negative Self; BCSS-PO, Brief Core Schema Scale-Positive Others; BCSS-PS, Brief Core Schema Scale-Positive Self; BDI, Beck Depression Inventory; BS-cog, cognitive part of Brooding Scale; BS emo, emotional part of Brooding Scale; BS-tot, total parts of Brooding Scale; EB, Externalizing bias of the IPSAQ; FNES, Fear of Negative Evaluation Scale; IPSAQ, Internal, Personal and Situational Attributions Questionnaire; PANSS-p, positive scale of Positive and Negative Syndrome Scale; PB, Personalizing bias of the IPSAQ; PsyD-emo, emotional part of Korean Version of the Psychotic Symptom Rating Scales-Delusion; PsyD-cog, cognitive part of Korean Version of the Psychotic Symptom Rating Scales-Delusion; PsyD-tot, total parts of Korean Version of the Psychotic Symptom Rating Scales-Delusion; R-GPTS-tot, total parts of the Revised Green et al., Paranoid Thought Scale (R-GPTS 1 assesses idea of reference, and R-GPTS 2 assesses idea of persecution)

Suppl Table 2. Severity of VR sickness symptoms.

| **Symptoms** |  | **After 1^st^ session** | | |  | **After 10^th^ session** | | |
| --- | --- | --- | --- | --- | --- | --- | --- | --- |
|  |  | **VR-control** | **VR-treatment** | ***p^a^*** |  | **VR-control** | **VR-treatment** | ***p^a^*** |
| General discomfort |  | 1.43±0.68 | 1.60±0.71 | 0.19 |  | 1.82±0.73 | 1.42±0.69 | 0.08 |
| Fatigue |  | 1.42±0.69 | 1.60±0.68 | 0.42 |  | 1.86±0.71 | 1.91±0.77 | 0.86 |
| Headache |  | 1.46±0.69 | 1.90±0.74 | 0.17 |  | 1.77±0.83 | 1.82±0.98 | 0.90 |
| Eye fatigue |  | 1.70±0.80 | 1.55±0.69 | 0.53 |  | 1.54±0.78 | 1.82±0.81 | 0.27 |
| Difficulty focusing the eyes |  | 1.71±0.86 | 1.85±0.86 | 0.59 |  | 1.78±0.88 | 1.58±0.79 | 0.54 |
| Increased salivation |  | 1.83±0.98 | 1.50±0.84 | 0.54 |  | 1.38±0.74 | 1.14±0.38 | 0.47 |
| Sweating |  | 1.00±0.00 | 1.36±0.52 | 0.26 |  | 1.33±0.52 | 1.33±0.58 | 1.00 |
| Nausea |  | 1.46±0.82 | 1.25±0.71 | 0.58 |  | 1.46±0.66 | 1.18±0.41 | 0.24 |
| Difficulty in concentrating |  | 1.53±0.74 | 1.48±0.81 | 0.83 |  | 1.60±0.83 | 1.71±0.77 | 0.71 |
| Head feels full |  | 1.50±0.71 | 1.73±0.80 | 0.46 |  | 1.67±0.90 | 1.69±0.86 | 0.94 |
| Blurry vision |  | 1.68±0.89 | 1.56±0.73 | 0.66 |  | 1.85±0.80 | 1.18±0.41 | 0.02 |
| Dizziness when opening eyes |  | 1.39±0.51 | 1.60±0.52 | 0.33 |  | 1.67±0.78 | 1.43±0.54 | 0.49 |
| Dizziness when eyes are closed |  | 1.33±0.71 | 1.40±0.52 | 0.82 |  | 1.63±0.92 | 1.56±0.53 | 0.85 |
| Dizziness with a spinning feeling |  | 1.33±0.62 | 1.47±0.64 | 0.57 |  | 1.79±0.58 | 1.62±0.77 | 0.52 |
| Feeling burdened by the stomach |  | 1.25±0.46 | 1.33±0.82 | 0.81 |  | 1.54±0.88 | 1.38±0.74 | 0.67 |
| Burp |  | 1.00±0.00 | 2.33±1.16 | 0.06 |  | 1.86±0.90 | 1.25±0.71 | 0.17 |

^a^ *t* test was performed to compare the severity of VR sickness symptoms between two groups.
Note: VR, Virtual reality.

Suppl Table 3**.** Direct, indirect and total effects of the final path models.

| **Dependent variable** | **Independent variable** | **Direct effect** | ***p*-value** | **Indirect effect** | ***p*-value** | **Total effect** | ***p*-value** | **SMC** |
| --- | --- | --- | --- | --- | --- | --- | --- | --- |
| 1) Model between the BCSS-NS and R-GPTS | | | | | | | | |
| R-GPTS | BCSS-NS | 0.01 | 0.97 | 0.33 | 0.01 | 0.33 | 0.02 | 0.39 |
|  | BDI |  |  | 0.23 | 0.05 | 0.23 | 0.05 |  |
|  | FNES | 0.62 | <.001 |  |  | 0.62 | <.001 |  |
| BDI | BCSS-NS | 0.44 | 0.001 |  |  | 0.44 | 0.001 | 0.19 |
| FNES | BDI | 0.37 | 0.01 |  |  | 0.37 | <.001 | 0.38 |
|  | BCSS-NS | 0.36 | 0.02 | 0.16 | 0.02 | 0.52 | <.001 |  |
| 2) Model between the BCSS-PO and R-GPTS | | | | | | | | |
| R-GPTS | BCSS-PO | -0.49 | <.001 |  |  | -0.49 | <.001 | 0.6 |
|  | BDI | 0.29 | 0.02 | 0.25 | 0.03 | 0.54 | <.001 |  |
|  | FNES | 0.47 | 0.01 |  |  | 0.47 | 0.01 |  |
| FNES | BDI | 0.53 | <.001 |  |  | 0.53 | <.001 | 0.28 |
| 3) Model between the BCSS-NS and PSYRATS-D | | | | | | | | |
| PSYRATS-D | BCSS-NS | -0.41 | 0.02 | -0.07 | 0.38 | -0.48 | 0.002 | 0.32 |
|  | BS-total | -0.31 | 0.08 |  |  |  |  |  |
| BS-total | BCSS-NS | 0.22 | 0.35 |  |  |  |  | 0.05 |

Standardized coefficient estimates are reported.

*Note.* BCSS-NS, Brief Core Schema Scale-Negative Self; BCSS-PO, Brief Core Schema Scale-Positive Others;
BDI, Beck Depression Inventory; FNES, Fear of Negative Evaluation Scale; R-GPTS, the Revised Green et al.,
Paranoid Thought Scale; SMC, Squared multiple correlations.

*
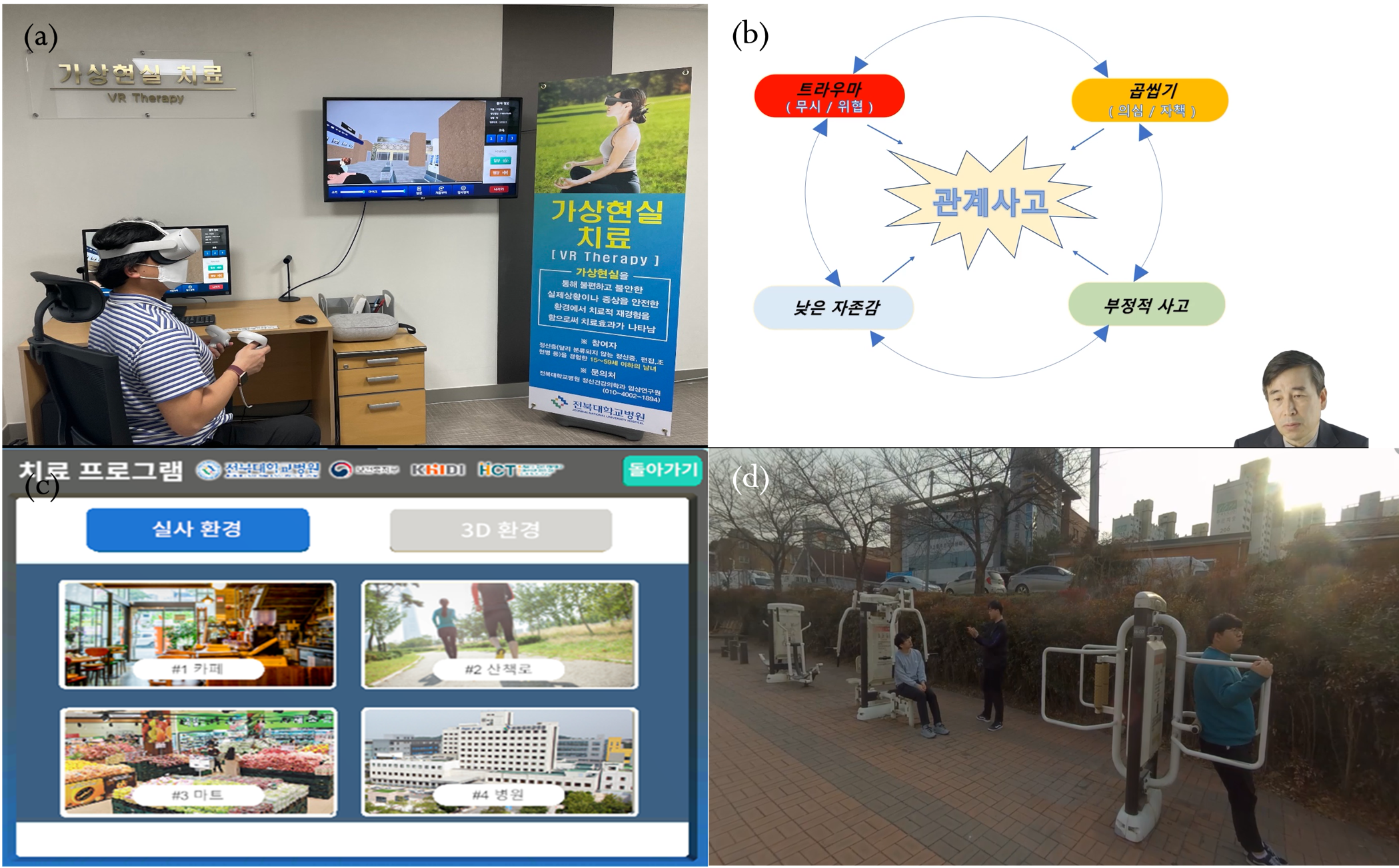
*


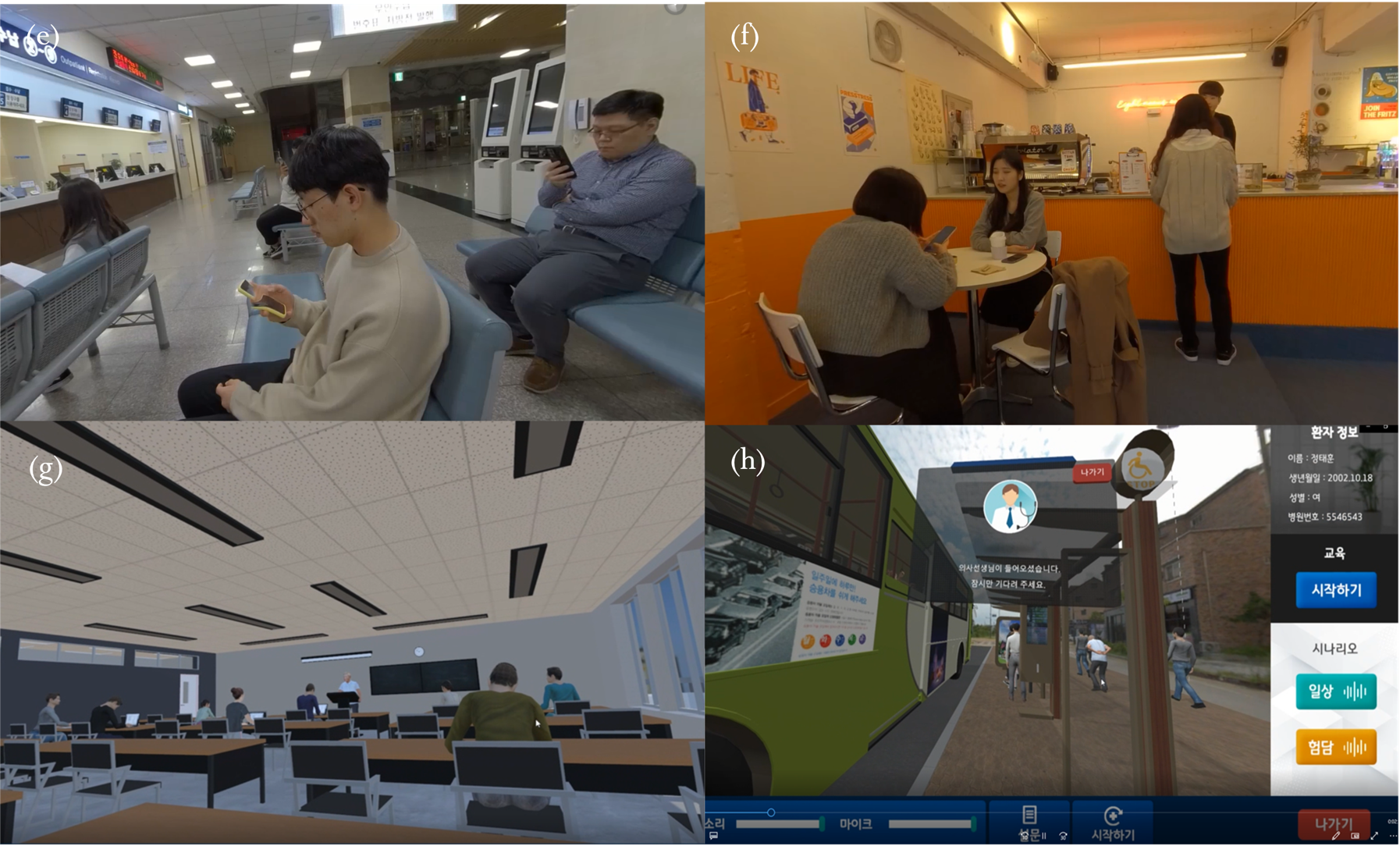


Suppl. Figure 1. Screenshots of contents of virtual reality (VR) therapy: (a) A scene of the person receiving VR therapy

in Digital therapy room; (b) Educational video; (c) Start-up screen of VR therapy program; (d) 360° 3D walking trails;

(e) 360° 3D hospital; (f) 360° 3D café; (g) Animated classroom; and (h) Animated bus station.

**Screened/assessed for eligibility (n=78)**

**Approached (n=90)**

**Randomized (n=78)**

**Allocated to VR-treatment (n= 38)**

**Allocated to VR-control (n= 40)**

**Analyzed (n= 32)**

**Analyzed (n= 38)**

**Excluded (n=12)**

- **Declined to participate (n=12)**

**Discontinued intervention (n = 6 )**

- **Discomfort due to eyeglasses**
- **Unable to wear the device due to head size**
- **Changed hospital**
- **Symptom worsening due to self discontinuation of medication**
- **Completed suicide**
- **Investigator-initiated termination**

**Discontinue intervention (n = 2)**

- **Decline to participate**
- **Dizziness**

**Excluded (n= 0)**

**Approach**

**Enrollment**

**Allocation**

**Follow up**

**Analysis**

Suppl. Figure 2. Consolidated Standards of Reporting Trials (CONSORT 2010) Flow diagram of the study procedure
